# The Choice of Pulmonary Autograft in Aortic Valve Surgery: A State-of-the-Art Primer

**DOI:** 10.1155/2021/5547342

**Published:** 2021-04-13

**Authors:** Francesco Nappi, Sanjeet Singh Avtaar Singh, Francesca Bellomo, Pierluigi Nappi, Adelaide Iervolino, Christophe Acar

**Affiliations:** ^1^Department of Cardiac Surgery, Centre Cardiologique du Nord de Saint-Denis, Paris, France; ^2^Department of Cardiothoracic Surgery, Golden Jubilee National Hospital, Glasgow, UK; ^3^Department of Clinical and Experimental Medicine, University of Messina, Messina, Italy; ^4^Department of Cardiovascular Sciences, Fondazione Policlinico Universitario A. Gemelli IRCSS, Rome, Italy; ^5^Department of Cardiac Surgery, La Pitié Salpetriere Hospital, Paris, France

## Abstract

The Ross procedure has long been seen as an optimal operation for a select few. The detractors of it highlight the issue of an additional harvesting of the pulmonary artery, subjecting the native PA to systemic pressures and the need for reintervention as reasons to avoid it. However, the PA is a living tissue and capable of adapting and remodeling to growth. We therefore review the current evidence available to discuss the indications, contraindications, harvesting techniques, and modifications in a state-of-the-art narrative review of the PA as an aortic conduit. Due to the lack of substantial well-designed randomized controlled trials (RCTs), we also highlight the areas of need to reiterate the importance of the Ross procedure as part of the surgical armamentarium.

## 1. Introduction

The use of the pulmonary autograft (PA) as a substitute for aortic valve and root replacement (AVR/AVRR) was initially proposed by Donald Ross, and subsequently, it has been the subject of large observational studies and randomized controlled trials (RCTs) [1–8].

The PA is relatively easily harvested and can be inserted without significant mismatch in size with the aortic valve and leads to few early site-related adverse events, one of all being endocarditis [9–16]. However, the medial layer of the PA is composed of a thin muscle layer, in contrast to a presence of a denser muscular layer and the elastic inner lamina of the aorta, which makes the PA more prone to expansion and adverse circumferential stress response, although more sensitive to adaptative remodeling [17–22].

Despite encouraging results, the use of the PA has been limited worldwide. This may reflect a resistance to change from the widely accepted gold standard of aortic valve replacement (which is performed using conventional mechanical or stented xenograft prostheses. Therefore, the implant of these most commonly used substitutes presents a short and familiar learning curve and at same time is easily reproducible by the vast majority of cardiac surgeons [23–28]. Furthermore, the current reluctance to use the PA may also be related to the need for advanced planning for its use, which is necessarily dependent on obtaining the patient's consent because potentially causing two pathologies with the intent of treating one [29–31].

Another point of heated discussion concerns the necessity to adapt the positioning of the PA in relation to the young age of the patient that is a perceived as a concern for the dilation of the neoaortic root and the consequent valve insufficiency leading for the dilation of the left ventricle. The latter requires additional surgical skills due to the involvement of the failed pulmonary autograft and homograft [32–34].

Finally, until recently, there has been a lack of persuasive evidence supporting a change in practice towards the extensive use of the PA as the ideal substitute for aortic valve disease. In this regard, the recent publications of large series evaluating the outcomes for more than 20 years on the use of the PA, either for the subcoronary or miniroot strategy by Sievers et al. and Nappi et al. [11, 35, 36], justify a review of the current evidence supporting the use of the PA for AVD as a superior alternative to conventional valves in selected categories of patients, its indications and contraindications, as well as specific technical aspects that might impact results.

## 2. Current Clinical Evidence

Large propensity-matched observational studies have shown that the average survival in patients in whom the PA was used as substitute to AVR is longer into the second postoperative decade after the Ross procedure [7, 35–43]. It is important to note that the statistical analysis of the majority of these studies has reported the survival was similar to that of the age- and sex-matched general population. Unlike these patient series managed with the Ross procedure, no other pertinent reports on aortic valve surgery performed in young and middle-aged adults provided results so relevant in terms of survival compared to the matched general population, including studies evaluating highly selected series of patients undergoing conventional AVR [44–48].

A comprehensive investigation that screened a large number of studies decidedly established the superiority of the Ross procedure [ 7, 11, 35–48]. Furthermore, in many reports, the survival of the population examined at 10-year follow-up was compared with that of the matched general population [7, 35, 36, 41, 42, 45, 46, 48], showing a superiority when comparing patients who received an AVR with the use of the pulmonary autograft than those who were managed with conventional bioprosthetic valves [7, 35, 36, 41, 42]. In five contemporaneous cohort studies [7, 35, 36, 41, 42], it was also noted that the Ross procedure had superior long-term clinical outcomes in the second postoperative decade.

On the contrary for 3 studies [45, 46, 48], both mechanical and bioprosthetic valves were associated with slightly higher long-term mortality compared with the matched general population when inserted in young and middle-aged adults. It is important to note that the best results were seen in large series of Ross procedures performed in experienced centers with long-term survival ranging from 87% to 95% at 15 years. Bucking rates of freedom from Ross-related reintervention were more variable ranging from 75% to 94% at 15 years [7, 11, 35–42]. Among other things, it is important to underline that most of the patients who received a Ross procedure in the large series had an age range between 34 and 44 years and hence amounting to only a 1% to 2% per patient-year reoperation rate. This percentage is very favorable and is not comparable to any conventional stented/nonstented biological valves. Importantly, Takkenberg et al. [49] revealed that patients who were managed with the Ross procedure had the additional benefit of low rate of long-term valve-related complications. This meta-analysis of observational studies reported low linearized rates of pulmonary autograft failure (0.78% per patient-year) and structural valve deterioration of pulmonary homograft (0.55% per patient-year). Endocarditis of pulmonary autograft and homograft was 0.26% and 0.20% per patient-year, respectively. Thromboembolism, bleeding, or valve thrombosis occurred with a rate of 0.36% per patient-year.

We are unaware of any randomized trials that have compared the Ross procedure to bioprosthesis AVR for severe aortic valve disease (S-AVD). However, evidence from one recent patient-level meta-analysis [25] strongly suggests that the surgery performed with the Ross procedure is beneficial. 872 unselected young adults (aged 17 to 40 yrs) underwent AVR in the United Kingdom between 2000 and 2012, and the patient population included the Ross procedure (26%), mechanical AVR (54%), and bioprosthetic AVR (17%). The study found a significantly higher event-free survival in patient who received the Ross procedure compared to those who were managed with the use of mechanical AVR. The latter group showed event-free that was superior to recipients of bioprosthetic AVR. When compared to the match general population, the Ross procedure has similar survival outcomes which were not demonstrable for either the mechanical AVR or the biological AVR groups [25]. It is important to emphasize that the great merit of this meta-analysis, although not a study level meta-analysis, is due to the statistical methodology that was used. The authors used a Bayesian dynamic survival model with a combination of propensity score matching, restriction matching, and stochastic augmentation to match patients from the 3 groups. The method undertaken allowed the correct evaluation of the three groups, eliminating most of the biases in examining the data from the National Congenital Heart Disease Audit of the United Kingdom, and was linked to the census of the Office of National Statistics to achieve long-term results [25]. Again, it is worth pointing out that data from observational studies suggest a benefit of mechanical AVR in the young population due to poorer outcomes for the bioprosthetic AVR. However, recently, a countertrend has emerged regarding the increased usage of bioprosthetic valves in young adult population with severe AVD [50, 51], which has opened up the possibility of the valve in valve TAVR procedure when a second intervention is required [52, 53]. Given the emergence of this new trend, a comparison could be of interest among a population of young adult patients who underwent the Ross procedure or bioprosthetic AVR.

A growing number of studies comparing recipients of the Ross procedure and other AVR options in adults have been performed. 1 RCT [7] enrolled 216 patients (mean age 39 years; mean follow-up 11 years; completeness of follow-up 97%) who received either the use of PA or Homograft to treat the aortic valve disease. The survival benefit associated to the use of the Ross procedure was significantly higher when compared to the use of the homograft. (95% vs. 78% at 13 years; hazard ratio: 0.22; *p* = 0.006). The effectiveness in using the PA to replace the aortic valve diseased is even more relevant if we consider that a large percentage of patients had received previous cardiac surgery and were managed with homograft aortic root replacements (42%) or underwent the Ross procedure for active endocarditis (8%). One of the most important statistics in this patient study was that the 13-year survival in recipients of the Ross procedure was uniform to that of the age- and sex-matched British general population [7]. Although differences in clinical outcomes for the Ross procedure are detected in one individual trial, a single RCT with a small number of patients raises concerns due to the sample size limitations.

Nowadays, there is a remarkable body of evidence to support the use of the Ross procedure, as it appears to offer an additional survival benefit over mechanical AVR. Indeed, more than 4 years ago, a propensity-matched cohort included 416 young and middle-aged adults with follow-up duration exceeding a mean of 14 years and reported a significant reduction in the hazard ratio (HR) for cardiac- and valve-related mortality in patients who received the Ross procedure (*n* = 208) compared to those who underwent conventional mechanical AVR (*n* = 208) (97% vs. 89% at 20 years; hazard ratio: 0.22; *p* = 0.03) [54]. This study revealed that early outcomes and overall survival were equal between arms and long-term freedom from reintervention was not different between the groups (87% in the Ross arm vs. 94% in the mechanical AVR arm at 20 years; hazard ratio: 1.86; *p* = 0.19). To note, firstly, 43% of recipients of the Ross surgery had aortic insufficiency preoperatively, and secondly, reoperations in this group included any surgical or percutaneous reintervention on the aortic and/or pulmonary position. Another important statistic significantly in favor of the Ross procedure was represented by higher freedom from thromboembolic and/or major hemorrhagic complications (99% vs. 80% at 20 years; hazard ratio: 0.09; *p* < 0.001) [54].

Another study from Buratto included 1928 patients undergoing isolated mechanical AVR and 392 with the Ross procedure, which were evaluated with a risk-adjusted analysis and with follow-up duration exceeding 25 years [55]. Among 275 propensity score-matched pairs, authors reported a significant reduction in mortality with the Ross procedure (94% vs. 84%; *p* = 0.018) whereas 30-day mortality was similar (Ross 0%; mechanical AVR 0.4%; *p* > 0.99) [55]. Importantly, no study based on large propensity-matched analysis has reported higher freedom from all-cause mortality with the Ross procedure versus mechanical AVR [56].

## 3. Harvesting

The Ross procedure is a valuable option to treat both congenital and acquired disease of aortic valve and the left ventricular outflow tract. The pulmonary autograft can be implanted using 2 methods: the subcoronary implantation or free-end technique [1, 3] and the root replacement procedure also known as the miniroot or full root technique [6]. In detail, pulmonary autograft explantation can involve two different types of pulmonary valve harvesting. In the subcoronary technique, the pulmonary valve is taken and inserted only with its leaflets and annulus; the noncoronary sinus of Valsalva can be retained for the Ross cylinder thereby providing a third possibility of implant [1, 3]. The subcoronary implantation is typically used in persons who have exceeded the somatic development [35]. The complete removal of the neighboring muscle and connective tissue to the annulus of pulmonary valve reduces the onset transvalvular gradient not bearable later [1, 2, 4, 35]. It is important to note that in most centers worldwide, the subcoronary implantation technique was left for multiple reasons, including sophistication of implant technique and the attractive option in implant of the root replacement position [6, 7, 11, 12, 37, 39–42].

In the miniroot technique, the pulmonary valve is transposed into the aortic position with its pulmonary trunk so that the PA is withdrawn from the infundibulum of the right ventricle, scrupulously respecting its morphology. The pulmonary infundibulum consists mostly of the conal or infundibular septum, which separates the pulmonary valve from the aortic and tricuspid valves. We still recognize a second part which is the anterior extension or division of the trabecula septomarginalis while the third, smaller part is a superior extension of the trabecular septum.

The miniroot technique is the most used procedure and included complete preservation of pulmonary valve and pulmonary trunk with the need to coronary artery reimplantation [6]. The insertion of the PA relative to the aortic annulus, which involves the positioning of the proximal suture, can be chosen according to the preferences of the surgeon, as well as the final length of the PA and the distal suture line. Although the protocol dictates the insertion of the PA on the annulus or below the annulus, scalloping of the muscle rim is always suitable. The advantage in favor of this surgical choice is that it is performed at the lower level to the valve leaflet that can reduce the risk of the onset of the gradient. The proximal suture line can be performed either with continuous or interrupted stich and can be reinforced with the use of a strip of pericardium or Teflon. The autograft root length can be different and is conditioned by the choice of the surgeon. The distal suture line can be performed at the lower level of sinotubular junction; nevertheless, many surgeons retain the entire length distally with a higher exposure to mechanical stress-strain phenomena [11, 15, 37–44].

Due to the different geometry and morphostructure of the aortic and pulmonary root (different commissural size and distribution), subcoronary implantation can be technically challenging, especially in patients with aortic insufficiency or bicuspid/unicuspid aortic valves. In addition, this approach is not possible when Ross's operation is the preferred operation for the congenital patients in the phase of pediatric growth. For this reason, the free-standing aortic root replacement (miniroot technique) has been the most used technique [34, 57].

The major concern with the use of pulmonary autograft used as miniroot implant is the increased risk of late pulmonary autograft dilatation due to the exposure of the full root to systemic pressures [33]. This dilatation may occur at the level of unsupported pulmonary sinuses, aortic annulus, and sinotubular dilatation with subsequent late autograft insufficiency. During the growth phase, Horer highlighted a different rate of increment of the PA root that was statistically relevant at level of neoaortic sinus (0.5 ± 0.1/year, *p* < 0.001) and the sinotubular junction (0.7 ± 0.2, *p* < 0.001), but not at the level of the annulus (0.1 ± 0.1, *p* = 0.59) at mean follow-up of 5.1 years [10, 11, 58].

To overcome the PA expansion and avoid potential risk of reoperation for autograft failure, a number of technical modifications have been proposed, but there are currently no standard recommendations for their use. Three different approaches are customary in higher volume center, but data that support the effectiveness on the long-term results of these techniques are lacking. The approach of inclusion of the PA within the patient's aortic root allows autograft to have a protection against the adverse effect of systemic pressure over time [40, 57]. Although the reduction of the size of the dilated aortic annulus could mitigate early dilation [11, 12], it did not prevent late failure which is likely due to preoperative aortic insufficiency [59]. More recently, the use of a reinforcement of the pulmonary autograft with external Dacron graft has been proposed to prevent late dilatation by a complete inclusion of miniroot in or partial slinging of the sinotubular junction only [60].

## 4. When to Use or Not to Use the Pulmonary Autograft

### 4.1. International Guidelines and Specific Directing of Professional Societies

However, concerns remain that any observational study is not without the potential risk of drawing skepticism for a surgical indication. In this regard, confusion can be generated in a form of selection bias that is independent of whether the elements provided in the study are aggregated data, propensity-matched, or processed with a multivariable analysis. It is logical to hypothesize that although the efficacy and safety of the Ross procedure can be partly explained by careful patient selection, it is more likely attributable to intrinsic characteristics of the PA that make it decidedly unique for the biomimetic characteristic of living tissue thus highlighting its hemodynamics and biological-adaptive behavior. Despite convergent evidence demonstrating superiority in long-term results with the use of the Ross procedure over the usage of other operation for AVR- including data from a randomized controlled trial [7], a systematic review and meta-analysis [ 49], as well as several large cohort studies with long-term follow-up- [11, 35–44, 54, 55] ESCTS, persist in omitting the Ross procedure as a surgical option as a Class IIb recommendation [61]. AHA/ACC guidelines recommend the Ross procedure as Class IIb Level C of evidence in patients who require a replacement of the aortic valve. Nevertheless, the use of pulmonary autograft must be performed by an experienced surgeon and may be considered for young patients when VKA anticoagulation is contraindicated or undesirable [7, 31, 61–64].

### 4.2. Indication and Contraindications

The Achilles heel of the PA replacing the AVR is the expansion of the vessel wall of the conduit after transposition into the left circulatory system where it is subjected to a higher arterial pressure, with a consequent decrease in the competence of the pulmonary valve and consequent increase in valve regurgitation [10–12, 32, 33, 58]. When it occurs, this entity can be seen on echocardiographic imaging and 3D CT scan [15, 19]. It is not unusual for this condition to negatively impact left ventricular function and clinical outcomes. Based on these pathophysiological concerns, current guidelines restrict the use of PA for patients with nonrepairable aortic valves. In planning the appropriate execution of the Ross operation, it is worth mentioning that although it has been used in an age group between two months and 80 years [6], when an isolated aortic valve repair or valve-sparing root replacement is indicated, it should be favored [65]. The optimal strategy for guiding the use of PA in aortic valve and/or aortic root surgery is to consider young or middle-aged patients under the age of 50 alongside those with otherwise nondisabling comorbid patients with aortic stenosis and a small or normal-sized aortic ring [66].

In young or middle-aged patients, the evidence has established that the Ross procedure offers a lasting solution, particularly in the female population [59], emphasizing in all the patient populations studied with the restoration of normal life expectancy with an excellent quality of life and a small number of valve-related complications. Rather, the management of anticoagulation requirements of a mechanical valve may constitute the major concern in women with future plan of pregnancy. In this direction, the use of biological substitutes relives the requirement for lifelong anticoagulation treatment [27, 28]. The use of the Ross procedure in this category of patients is to prevent the prolonged administration of anticoagulant drugs, with the consequent benefit of avoiding the continuous risk of valve thrombosis, thromboembolism, and bleeding. Evidence published in cohort studies including a large population of patients with >20 years of follow-up who were chronically anticoagulated reported a linearized rate of thromboembolic complications or major bleeding ranging from 1.1% to 4.5% per patient-year in patients who were managed with the use of mechanical prostheses for AVR [67, 68].

Although advances in prosthetic manufacturing have allowed the use of new-generation mechanical valves, it is potentially less thrombogenic, and lower international normalized ratio targets are required; however, self-monitoring of oral anticoagulants is still necessary [69, 70]. There is no doubt that the risk of thrombotic and haemorrhagic complications has been reduced, but these remain an unavoidable drawback for the use of mechanical valves.

From the results provided in the population of patients aged between 50 and 60 years, it is evident that the Ross procedure finds its optimal indication in people who, due to the absence of obvious comorbidities, are certainly more likely to benefit from the use of the pulmonary autograft. These patients had specific demographic, morphological characteristics and the absence of previous pathologies in their clinical history. In this respect, they had an expected life expectancy of 15 years, an active lifestyle, favorable anatomy, the absence of other major concomitant heart disease, and poor comorbidity.

Contraindications to the use of the Ross procedure are preoperative aortic regurgitation, an aortic ring of ≥27 mm, and pulmonary size mismatch [11, 15, 59]. These specific characteristics put the duration of PA at risk by leading for progressive dilation of the aortic annulus due to its preexisting morphostructure. Depending on the surgical method used to strengthen the aortic annulus and described in the PA harvesting, it is possible to mitigate the effects of pressure stress on the connective tissue alterations, thus making a Ross procedure possible even in the presence of a nonoptimal anatomical substrate. For example, the full inclusion of PA is not uncommon in patients with a tendency to progressive dilatation of the implanted conduit or those with poorly controlled blood pressures that may be too high and disabling for the PA [1–6, 8, 40, 43, 57]. However, these patients who exhibit disorders of the connective tissue structure can potentially have a reduced benefit from use by the Ross procedure because once the PA is encased in the rigid dacron prosthesis, its mobility is greatly reduced with a detrimental effect on the function of the PA as a living tissue [18–20, 60]. For these recipients of the Ross procedure, although not ideal candidates and for whom the use of PA is not discouraged, implant failure may occur, as shown with the reoperation rate ranging from 20% to 50%—still considered low—and between 1% and 2% per patient-year [37, 41, 49].

What are then the modern indications for the use of the Ross procedure for AVD, and what do the recent studies add to our knowledge? We believe that the use of PA should be considered when planning any aortic valve and root surgery, given its characteristic of living tissue that corresponds to somatic growth, low risk of dimensional mismatch, versatility in achieving efficacy and safety for any type of operation on the aortic valve and on the aortic root, its perioperative safety, comparable to conventional mechanical and biological valves, and its late duration as a valve substitute, now clearly demonstrated, superior to traditionally used prostheses. Even the presence of a pathological bicuspid aortic valve today cannot be considered a contraindication. The study by Poh et al. [71] reported favorable results in a consecutive series of 129 patients (mean age 35 years; mean follow-up 10 years; follow-up completeness 98%) who had a bicuspid aortic valve (BAV) and pure aortic insufficiency. In these recipients of the Ross procedure, a freedom from reoperation 20 years and/or aortic insufficiency greater than mild was 85% at 20 years, thus emphasizing that even patients with preoperative aortic insufficiency, who received the use of pulmonary autograft adding the reinforcement variant such as the inclusion cylinder (Ross cylinder), could have lasting outcomes over time [1–6, 40]. Therefore, given the recent findings on the time span of prosthetic AVRs, the use of Ross procedure in young patients even when it is achieved in the presence of aortic insufficiency may still be the best option. Of note, a nonindicated criterion for PA harvesting in Ross's operation has been shown in patients with familial aortopathy or connective tissue disease because delayed dilation and significant autograft failure occurs in this population. However, it should be noted that the population with BAV and with no hereditary aortopathy or connective tissue disease do not reveal any red flag contraindications for receiving the Ross procedure [72]. Compared to the alteration without BAV, several studies have recently associated stronger evidence of a potentially higher risk of autograft dilation in recipients of the Ross procedure with BAV which may allow for greater variability in terms of good late outcomes [73, 74]. Furthermore, the use of PA does not appear to increase the risk of reoperation in the population of patients with bicuspid aortic valve compared to those with a tricuspid valve morphology. There is significant overlap between the biomechanical behavior of the bicuspid aorta and tricuspid aorta. In fact, when we observe the survival and the reoperation rate, we note that it does not vary with respect to the phenotype and that the results at 19 years provided by centers with large series and experience did not report substantial differences [72]. Although some groups used the PA in congenital aortic valve disease and with a BAV predominantly (50% to 90%), data from the interaction between these baseline characteristic and rates of late aortopathy or dissection were extremely low in large series with long-term follow-up [7, 25, 36, 38, 39, 54], and therefore, its use is not discouraged. Furthermore, there is a concern related to BAV disorder which tends to be heterogeneous and can manifest itself in a small subgroup of patients with an associated inherited aortopathy sometimes as noted by concomitant annuloaortic ectasia and aortic insufficiency. Such a condition deserves counter-condemnation for the Ross procedure.

Without doubt, the option for Ross procedure, although performed with an external reinforcement, is indicated in young patients with BAV and an aorta with a maximum diameter of 40 mm who do not have a hereditary pathology of the connective tissue or of the aorta. As reported below, the condition for the choice of the PA as substitute is conditioned by the use of surgical techniques that allow modified Ross procedures with the use of various external supports aimed at stabilizing the sinotubular junction and thus minimizing the risk of late aortic insufficiency and PA dysfunction. Overall, the choice of PA and surgical strategy for Ross procedure is based in part on evidence of comorbidities, ethical consideration, skill, and experience. To optimize outcomes in the population receiving the use of the Ross procedure and avoid the autograft failure, some considerations are needed on the knowledge of coexisting conditions that limit life expectancy to <15 years such as chronic dialysis in renal failure or radiation-induced valve disease. Also, some autoimmune diseases, for example, lupus erythematosus or rheumatoid arthritis, can raise concerns about the functional duration of the PA. Unquestionably, today, we have gained adequate knowledge of when to choose the Ross procedure and when it is deemed appropriate for the right patient and how to perform it perfectly. For example, the data provided by the current literature do not identify the use of PA as an appropriate choice in patients with rheumatic disease [75]. On the other hand, due to the low rate of relapse, the use of the Ross procedure is suitable for patients with aortic valve endocarditis [15, 16, 76, 77].

The concern related to the use of pulmonary autograft in aortic position is the potential long-term failure of the 2 valves, aortic and pulmonary. For many surgeons, this represents the Achilles heel of the Ross procedure and can certainly be deterrent to its widespread use. In fact, a patient who initially exhibits single-valve disease may subsequently require repeat surgery to treat two diseased valves. As noted by Stulak et al. [30], when choosing the pulmonary autograft in the Ross procedure, the use of biological substitutes raises some ethical questions about the chances of the procedure failing or the need for reinterventions which are not uncommon.

For this reason, the problem of the longevity of the biological substitute is a priority, trying to give reasonable and favorable solutions to avoid adverse complications. Primary leaflet failure and dilation of the annulus, Valsalva sinuses, or sinotubular junction may occur in the patient who has an indication for a second operation after a Ross procedure for pulmonary autograft failure [33, 78]. Several studies have reported a very high and hardly acceptable rate of reoperations for pulmonary autograft expansion [29, 30, 33]. It is important to underline that in recipients of Ross procedures who experience the PA dilation leading to aortic insufficiency, the increase in the diameter of the neoaortic root occurs at the time of discharge from the hospital, suggesting the existence of technical problems related to the procedure [11, 12, 18–21]. Surgeons with more experience in using the Ross procedure have pointed out that technical improvements reduce the risk of pulmonary autograft dilation. Variations to the two standard implantation techniques have demonstrated efficacy and safety of PA use with excellent long-term results [33, 40, 57, 60], such as suturing the pulmonary autograft in an intra-annular position. With this approach, the native aortic annulus is allowed to support and stabilize the neoaortic root, provided that there is no preoperative dilation of the native aorta or annulus [ 11, 15].

Associated with autograft failure, there is a risk of pulmonary homograft dysfunction which is used to reconstruct the ventricular-pulmonary outflow tract. The failure of pulmonary homograft is mainly manifested by the onset of an increasing valvular and supravalvular pulmonary stenosis which occurs more frequently at the level of the distal anastomosis and appears to be linked to an inflammatory activity [79]. Instead, pulmonary insufficiency is due to a homograft cusp prolapse and occurs in a smaller percentage of cases [ 78, 80]. A risk factor that can accelerate dysfunction is degeneration of the pulmonary homograft and preoperative pulmonary hypertension, especially when it is severe and/or irreversible. It is important to underline that dysfunction in the pulmonary homograft rarely leads to a life-threatening issue, because the volume and/or pressure overload of the right ventricle is generally endured for a long time before requiring repeat surgery [11, 15]. In new platforms for the treatment of structural heart disease, the patient presenting with a pulmonary homograft failure is treated with a percutaneous access using the THV procedure [81]. The new armamentarium available in market is the Melody valve (Medtronic, Dublin, Ireland) [82] or the Sapien system (Edwards Lifesciences, Irvine, California).

Evidence based on follow-up of patients (*n* = 212) included in the Toronto series [37] demonstrated that choosing an oversized pulmonary homograft (25 mm are generally not recommended) reduced the risk of homograft failure. Although the authors reported 93% of patients not needing reoperation at 20 years, different degrees of pulmonary homograft dysfunction were detectable on echocardiography. Therefore, they concluded that the possibility for these patients to undergo repeat surgery in the future was not negligible [37].

The ideal substitute to use for the reconstruction of the right ventricular-pulmonary trunk has been the reason of passionate discussion because the choice of the conduit can decisively affect its durability. Evidence shows that the duration over time of the pulmonary autografts is greater than that of the aortic homografts when they are implanted in the pulmonary position [79, 80]. We reported the absence of pulmonary homograft failure in patients with Ross procedures at 23 years of follow-up [11, 15].

The choice of using cryopreserved pulmonary homografts was preferred for a long time because homografts were considered the ideal available substitutes for right ventricular outflow tract reconstruction [83]. More recently, the use of decellularized pulmonary homografts is established on the cardiac surgery scenario with growing interest [84]. However, to consolidate their use, it is necessary to obtain longer follow-ups that can demonstrate whether the decellularized derivatives will reach a longer duration than the cryopreserved homografts [85] [86]. Another alternative for right ventricular outflow tract reconstruction is the use of stentless xenograft roots such as the Freestyle Porcine Aortic Root (Medtronic) [87], although the use of this conduit in the pulmonary position is not supported by long-term data on duration.

.

## 5. Ross Experimental Studies

**Figure 1:**
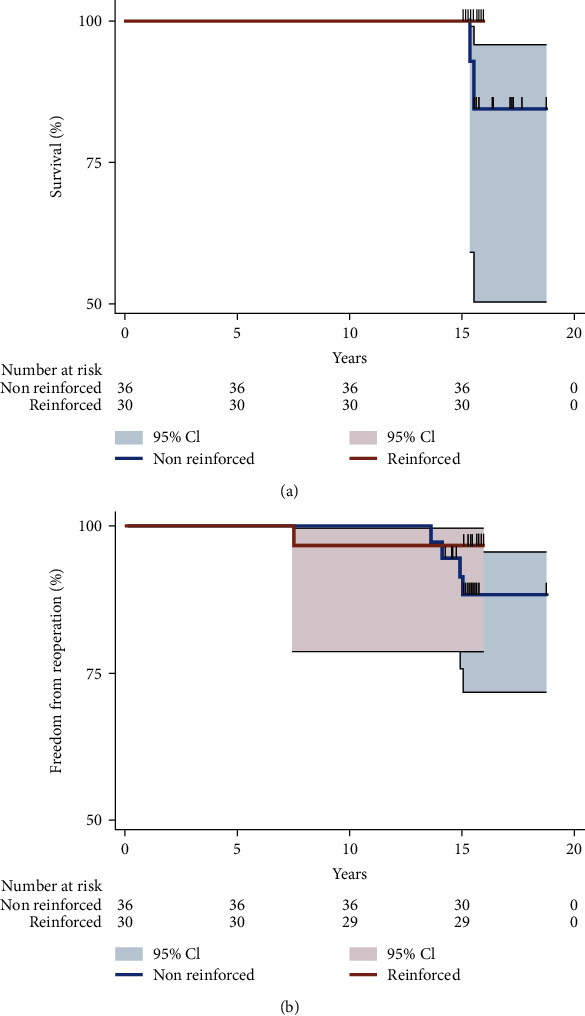
(a) Survival of the reinforced vs. nonreinforced Ross procedure. (b) Freedom from reoperation of the reinforced vs. nonreinforced Ross procedure. Reproduced with permission from Nappi et al.

We evaluated the effect of reinforced and nonreinforced Ross procedure on long-term echocardiographic outcomes in 66 patients who received a PA for aortic valve surgery. The results are clearly delineated in Figure 1 which shows both the survival and freedom from reoperation. The 36 patients undergoing nonreinforced Ross reported the mean increase in diameters of 1.28 ± 0.38 mm (3.9%) at the annulus level (compared with reinforced, *p* = 0.001) and 3.95 ± 0.64 mm (12.1%) in the nonreinforced group at the Valsalva sinus level (compared with reinforced, *p* = 0.001) [11].

The experimental project Ross is a European alliance of investigators who aim to provide the basis for studying how to prevent the expansion of pulmonary autograft used in aortic valve surgery. The project was initiated in January 2011 and is achieved with collaboration of the Department of Cardiac Surgery of Centre Cardiologique du Nord and la Pitie Salpetriere Hospital. The first results were presented and discussed in June 2013 at the annual meeting of the Heart Valve Society in Venice, Italy [87–89].

The primary objective of Ross's experimental project was to combine the individual data of the experimental animal model by comparing nonreinforced and reinforced pulmonary autograft as ideal substitutes for aortic valve surgery. Using an experimental model of growing sheep based on the simulation of the Ross operation, the experimental project Ross has estimated that the analysis of the results would have detected significant differences in the pulmonary autograft morphostructure at 6-month follow-up. The pulmonary autograft was inserted in the descending aorta while the right ventricle outflow tract was reconstructed with a fresh homograft from another lamb of the same age and weight or native pericardial neoconduit [84, 90].

To date, in all the reported studies, the comparison between reinforced and nonreinforced pulmonary autograft did not consider the somatic growth variable which is fundamental when the Ross operation is performed during the patient's growing age. Echocardiographic findings were largely undersized to assess differences in clinical events. Although the rate of superior expansion of the nonreinforced pulmonary autograft was established, there was no available evidence on any potential clinical benefit for the late outcomes.

**Figure 2:**
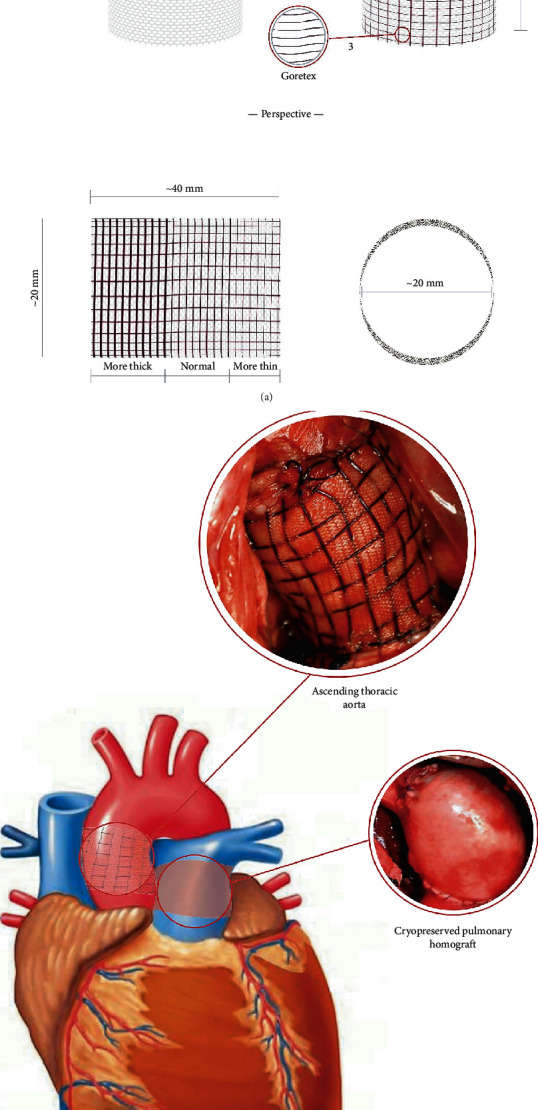
(a) Semiresorbable cross-linked prosthesis composed by two layers: (1) resorbable polydioxanone, (2, 3) nonresorbable expanded polytetrafluoroethylene. (b) The prosthesis is used to reinforce the implanted pulmonary autograft that replaced the diseased aortic valve. The right side of the heart is reconstructed with a pulmonary homograft.

The results were presented and discussed in June 2013 at the annual meeting of the Heart Valve Society in Venice, Italy [87]. At 6 months, the animal weight was doubled (27 ± 5 kg at day 0, and 55 ± 10 kg at 6 months), suggesting a normal growth process. At 6 months, there were no significant differences in expansion of PA between nonreinforced Ross and reinforced with external nonresorbable polyester (20 ± 1 mm vs. 19 ± 2 mm; index ratio, 1.05; *p* = .4) but with the exponential increase in the expansion of pulmonary autograft (42%) in reinforced Ross with bioresorbable vascular scaffold (BVS/polydioxanone) and with semibioresorbable vascular scaffold, which combine the polydioxanone (PDS) and expanded polytetrafluoroethylene (e-PTFE) (28 ± 2 mm vs. 19 ± 2 and 27 ± 2 mm vs. 19 ± 2; index ratio 1.42, respectively). These are depicted in Figures 2(a) and 2(b) which show the prosthesis Figure 2(a) and its appearance post implantation Figure 2(b). In the reinforced Ross with semibioresorbable vascular scaffold, the PA behaved similarly to the normal aorta in the growing lamb. This was the first time in the history of Ross operation that stress shielding, growth, and remodeling of the pulmonary autograft were studied by a mathematical and biomechanical analysis leading to a better understanding of the biological potential of the pulmonary autograft [86, 90].

## 6. The Biological Potential of Pulmonary Autograft for Limiting the Adverse Event

The use of pulmonary autograft in aortic valve surgery preserves, as living tissue, the structural and functional unity of the neoaortic root favoring better long-term clinical outcomes. The PA offers a continuous mediated living tissue activity that cannot be ensured by any other valve substitute with nonliving tissue characteristics. Although homografts have shown long-term viability, from a mechanical standpoint, they have proven to be inferior to pulmonary autografts [1–6, 91]. Vesely et al. [92] demonstrated that the pulmonary infundibulum, when placed in the aortic position, has an additional 30% potential for distensibility in respect to the native aortic root, allowing for a considerable degree of distortion without causing valve insufficiency.

The increased distensibility of viable pulmonary autograft is associated with recognized adaptive remodeling when the PA is transposed to an aortic position under systemic loading, thus mimicking the highly sophisticated anatomy and function of the native aortic root [86–91]. The endothelial and interstitial valvular cells intervene in the remodeling process when the PA is subjected to action of systemic pressure by means of the EphrinB2 expression [91]. This marker, which is present on the endothelium of the heart valves in the left side but not in the right side of the cardiac structure, leads to the remodeling of the extracellular matrix with an increased smooth muscle actin production [93].

We have enhanced the remodeling capacity of the PA and at the same time reduced the negative effect of systemic pressure on the vessel wall using a semibioresorbable vascular scaffold that combined the polydioxanone to expanded polytetrafluoroethylene (BVS/PDS-e-PTFE) [86, 90, 93, 94]. The use of nonresorbable polyester reinforcement, as suggested in the literature and also demonstrated in our experimental model of Ross operation, can greatly affect both the viability of the tissues due to the inflammatory process of foreign body reaction and the biomechanical characteristics of the reinforced PA [95–100]. We have demonstrated the presence of macroscopic and microscopic alterations from within the explanted graft. The nonresorbable polyester mesh was found to be visible and had partly migrated through the PA wall as highlighted with histochemical analysis [86, 90, 94, 95].

We have shown that the interaction between temporary bioresorbable reinforcement and pulmonary autograft has orchestrated a complex vascular remodeling process based on a balance between inflammation and production of extracellular matrix resulting after biomaterial resorption, in a “neovessel” which has characteristics similar to the aorta but is still biologically alive and capable of growing. The use of resorbable polyester was also associated with higher production of new extracellular matrix that was mainly characterized by a higher content of elastin fiber in the PA, as well as by a more compact organization of collagen fibers in the elastic zone of the vessel. Interestingly, the metalloprotease MMP-9 was found to be overexpressed indicating an ongoing matrix remodeling process. In parallel, cell proliferation was found to be increased in this group as testified by the significantly higher percentage of Ki67-positive cells (26.89%, 68.4% in the nonreinforced vs. 51.55%, 69.7% in the reinforced group, *p* < 0.05). These findings were coupled with a significant reduction in apoptosis in the reinforced PA supporting the idea of an active remodeling process in this group (47.8% ± 7.2% in the nonreinforced Ross vs. 17.5% ± 5.1% in the reinforced group, *p* < 0.05) [93].

These findings offered a plausible biologic and biomechanical explanation to the observed advantage in clinical outcomes. A biocompatible reinforcement of the PA would therefore allow induction of an in vivo creation of a PA with morphostructural characteristics that allow improved tolerance to the hemodynamic load of the arterial system and to guarantee a harmonic increase in size during somatic growth [18, 94, 95, 101, 102].

**Figure 3:**
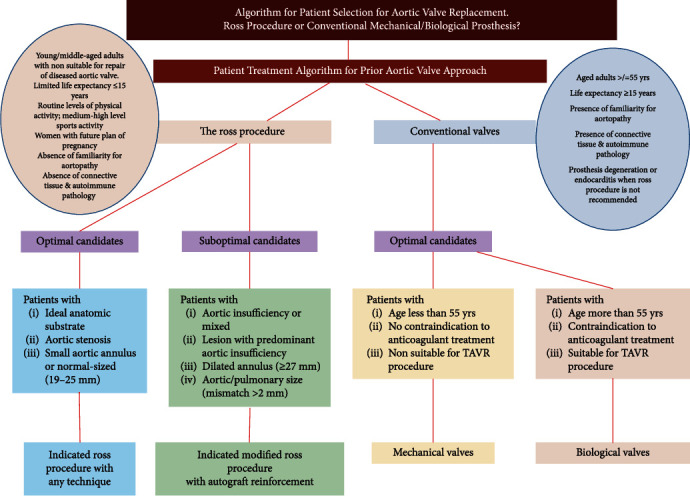
Algorithm for patient selection for aortic valve replacement. Ross procedure or conventional mechanical/biological prosthesis.

To further guide the choice of valve selection, we have formulated an algorithm to guide clinicians based on the evidence available as shown in Figure 3.

## 7. Conclusion

Although the impact of the Ross procedure on long-term survival has been proven in young and middle-aged adults with significant impact when matching that of the age- and sex-matched general population, large propensity-matched observational studies and meta-analysis failed to cement its place as the ideal aortic valve substitute. The use of pulmonary autograft in aortic valve surgery has provided solid evidence of better long-term freedom from death and valvular complications compared to other conventional aortic valve prostheses used for AVR. Evidence has suggested that the Ross procedure provides better results when performed in centers of excellence where high volumes of aortic root surgery are performed. Much has been learned in the past 50 years of the Ross operation practice about the use and behavior of pulmonary autograft transposed in aortic position, but we are still in the process of iterative learning with more research needed. Unfortunately, there is a lack of evidence due to the absence of well-designed RCTs comparing the Ross procedure to mechanical or biological valves. Furthermore, greater confirmatory results can be provided by randomized clinical trials with the use of biocompatible external reinforcements able to stimulate, guide, and improve the natural processes of biological remodeling of the graft and reaction to foreign materials while respecting tissue growth. These studies could be a turning point in solving some of the drawbacks of the Ross procedure. Nevertheless, a significant impulse can be given by the biomechanical studies performed on the heart valves and on the extracellular matrix with the application of finite element analysis [103–112].
